# 2401. The Role of Quantitative Microbial Cell-free DNA (mcfDNA) Sequencing (Seq) from Plasma in the Diagnosis (Dx) of Staphylococcal Cardiac Implantable Electronic Device Infective Endocarditis (CIED IE)

**DOI:** 10.1093/ofid/ofad500.2021

**Published:** 2023-11-27

**Authors:** Adolf W Karchmer, M Jenifer Kaufman, Ahmed Abdul Azim, Polly van den Berg, Jason D Matos, Daniel B Kramer, Nicholas R Degner, Peter Zimetbaum

**Affiliations:** Beth Israel Deaconess Medical Center, Boston, Massachusetts; Beth Israel Deaconess Medical Center, Boston, Massachusetts; Rutgers Robert Wood Johnson Medical School, New Brunswick, New Jersey; University of Pennsylvania, Philadelphia, Pennsylvania; Beth Israel Deaconess Medical Center, Boston, Massachusetts; Beth Israel Deaconess Medical Center, Boston, Massachusetts; Karius Inc., San Francisco, California; Beth israel deaconess medical center, Boston, MA

## Abstract

**Background:**

Optimal treatment (rx) of staphylococcal CIED IE requires removing the entire CIED. Difficulty of dx plus cost and potential morbidity of rx make treatment challenging. Complicated staphylococcal bacteremia is associated with prolonged detection of plasma mcfDNA during antibiotic rx. To assess for IE, we studied plasma mcfDNA kinetics in CIED patients (pts) with staphylococcal bacteremia undergoing CIED removal.

**Methods:**

With written consent, CIED pts (no persistent confounding infection) with bacteremia in the prior ≤ 7 days (d) and planned CIED removal had serial plasma specimens collected [Figure]. Quantitative mcfDNA-Seq was done using the Karius Test^®^ at Karius CLIA certified Laboratory (Redwood City,CA) on batched pt specimens. Clinical data were obtained from the electronic medical record. CIEDs were removed through sterile pockets or surgically except in pt 012. Definite CIED IE was defined by multiple d of bacteremia **plus** a definite lead / tricuspid vegetation per echocardiogram, direct inspection or lead tip culture; multiple d bacteremia **or** a definite vegetation defined possible CIED IE.

**Figure:**
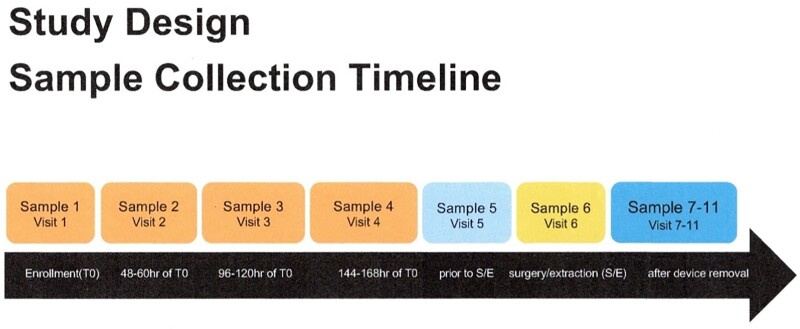

**Results:**

Nine pts with definite CIED IE had bacteremia 3-13 d (median 6 d) with *Staphylococcus aureus* (7 pts) or *S. epidermidis* (2 pts: 012 who due to retained lead fragment relapsed after rx 021) [Table 1]. At consent, after 2-11 d rx (median 6 d) mcfDNA was detected in 8 of 9 pts; in all 9, on CIED extraction mcfDNA increased significantly from consent and pre-extraction levels [Table3]. Five pts with possible *S.aureus* CIED IE had 1-3 d (median1 d) bacteremia [Table 2]. None had plasma mcfDNA after 5 d rx and 4 of 5 had no mcfDNA detectable on device extraction [Table3]. McfDNA patterns in definite CIED IE (8 of 9 pos on consent / pre-extraction and had an increase on extraction) differed from those in possible CIED IE (4 of 5 neg at 5 d of rx and none detected on extraction) [p=0.023 Fisher Exact].

**Figure:**
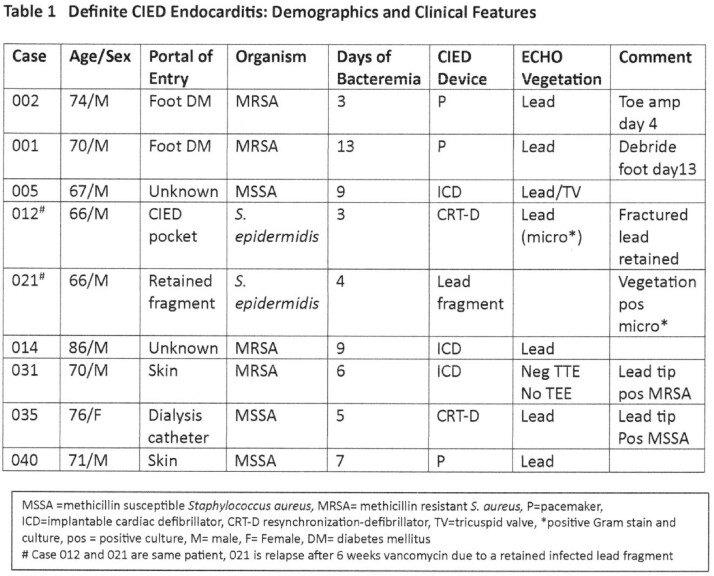

**Figure:**
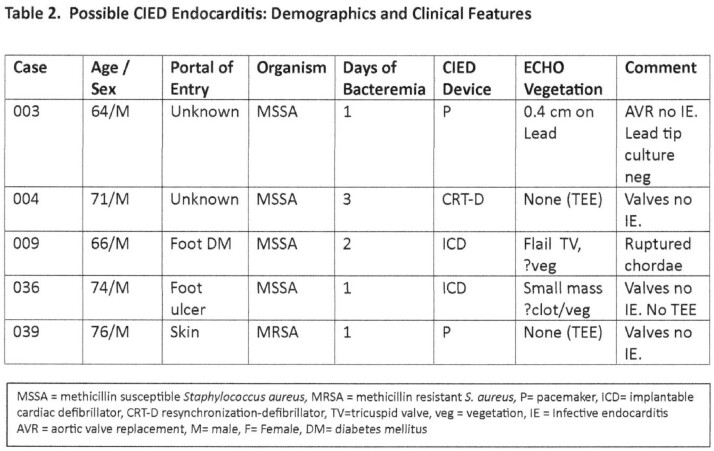

**Figure:**
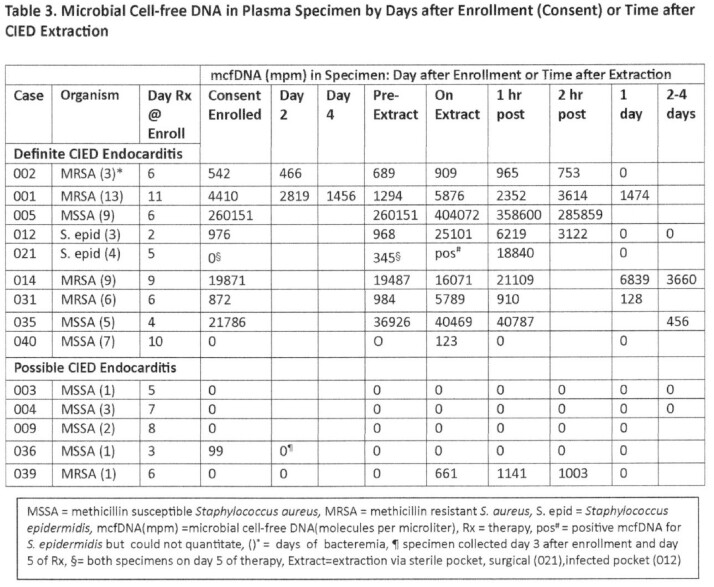

**Conclusion:**

Quantitative mcfDNA patterns can help distinguish definite from possible CIED IE and absence of mcfDNA in plasma of possible CIED IE on device extraction suggests that the CIED may not be infected. After 5 days of rx, plasma mcfDNA-Seq in CIED pts with staphylococcal bacteremia coupled with clinical features may identify patients that can be effectively treated without device removal - a testable hypothesis.

**Disclosures:**

**Adolf W. Karchmer, MD**, DeBiopharma: Advisor/Consultant|DeBiopharma: Honoraria|Karius: Grant to Institution for this study **Jason D. Matos, MD**, Merck: Wife is an employee **Nicholas R. Degner, MD, MPH, MS**, Karius: Employer

